# Patient-to-patient transmission of hepatitis C virus (HCV) during colonoscopy diagnosis

**DOI:** 10.1186/1743-422X-7-217

**Published:** 2010-09-08

**Authors:** Fernando González-Candelas, Silvia Guiral, Rosa Carbó, Ana Valero, Hermelinda Vanaclocha, Francisco González, Maria Alma Bracho

**Affiliations:** 1 Centre Superior d'Investigació en Salut Pública (CSISP), Àrea de Genòmica i Salut, Conselleria de Sanitat, Generalitat Valenciana, València, Spain; 2 Direcció General de Salut Pública (DGSP), Àrea d'Epidemiologia, Conselleria de Sanitat, Generalitat Valenciana, València, Spain; 3 Institut "Cavanilles" de Biodiversitat i Biologia Evolutiva (ICBiBE), Universitat de València, València, Spain; 4 Centro de Investigación Biomédica en Red de Epidemiología y Salud Pública (CIBERESP), Ministerio de Ciencia e Innovación, Spain; 5 Microbial Pathogenesis Unit, Centre for Infectious Diseases, University of Edinburgh, UK

## Abstract

**Background:**

No recognized risk factors can be identified in 10-40% of hepatitis C virus (HCV)-infected patients suggesting that the modes of transmission involved could be underestimated or unidentified. Invasive diagnostic procedures, such as endoscopy, have been considered as a potential HCV transmission route; although the actual extent of transmission in endoscopy procedures remains controversial. Most reported HCV outbreaks related to nosocomial acquisition have been attributed to unsafe injection practices and use of multi-dose vials. Only a few cases of likely patient-to-patient HCV transmission via a contaminated colonoscope have been reported to date. Nosocomial HCV infection may have important medical and legal implications and, therefore, possible transmission routes should be investigated. In this study, a case of nosocomial transmission of HCV from a common source to two patients who underwent colonoscopy in an endoscopy unit is reported.

**Results:**

A retrospective epidemiological search after detection of index cases revealed several potentially infective procedures: sample blood collection, use of a peripheral catheter, anesthesia and colonoscopy procedures. The epidemiological investigation showed breaches in colonoscope reprocessing and deficiencies in the recording of valuable tracing data. Direct sequences from the NS5B region were obtained to determine the extent of the outbreak and cloned sequences from the E1-E2 region were used to establish the relationships among intrapatient viral populations. Phylogenetic analyses of individual sequences from viral populations infecting the three patients involved in the outbreak confirmed the patient pointed out by the epidemiological search as the source of the outbreak. Furthermore, the sequential order in which the patients underwent colonoscopy correlates with viral genetic variability estimates.

**Conclusions:**

Patient-to-patient transmission of HCV could be demonstrated although the precise route of transmission remained unclear. Viral genetic variability is proposed as a useful tool for tracing HCV transmission, especially in recent transmissions.

## Background

HCV is predominantly transmitted by the parenteral route in procedures such as unscreened blood transfusions, injections related to intravenous drug use (IDU), injections related to health-care procedures, invasive medical and surgical interventions and, to a lesser extent, other percutaneous exposures [1-3]. Perinatal or sexual transmissions are considered far less efficient than transmission through large or repeated parenteral exposure [1,4-6]. Current HCV prevalence worldwide is far from even [4,5] and is expected to fluctuate according to both availability and adherence to prevention measures at health-care facilities and also to changes in IDU-related habits [5]. HCV prevalence, estimated to be about 3% in 1999 [7], is mainly due to past infected blood transfusions and past and present injected drug use. Due to the asymptomatic nature of most HCV infections, the epidemic remains largely unnoticed with most chronic cases having been infected years ago, and with relatively few cases of acute infections being reported [8].

Interestingly, risk factors accounting for infection remain unknown in 10-40% of the patients with acute or chronic hepatitis C [6,9]. In contrast to chronic hepatitis, cases of recent HCV infection could shed greater light on mechanisms of HCV transmission because the source of infection can be traced more accurately [8]. HCV infection associated with medical procedures, which are still unrecognised or underestimated, is an issue of great concern [8,10]. A nosocomial HCV mode of transmission that remains controversial is diagnostic or therapeutic digestive endoscopy (gastroscopy and colonoscopy). These infection routes are not even mentioned in recent reviews specifically dealing with the epidemiology of HCV infection [4,5], although they receive varying attention in others [3,6].

The first patient-to-patient hepatitis C transmission through colonoscopy was reported by Bronowicki *et al*. in 1997 [11]. Since then, a full consensus has been reached about the relationship between strict adherence to standard safety measures and null risk of transmission via endoscopy or endoscopy-related procedures [12,13]. However, further doubts have been casted on the true extent of HCV transmission via these procedures, ranging from rarely reported [13] to underestimated [14]. Two case-control studies carried out in France reported significant association between HCV infection and endoscopy procedures [15,16]. These studies showed that no significant decrease in this endoscopy-associated risk was found between 1980 and 1999 [15], and that HCV was still transmitted in France through specific invasive procedures including digestive endoscopy, at least, until 2002 [16].

A problem associated with identifying the source of nosocomial HCV infection is that retrospective transmission studies often fail to identify the exact procedure causing infection [10,14]. Hence, only well-documented case reports contribute to providing evidence for this particular HCV transmission in healthcare units. In two recent studies of patient-to-patient HCV transmission in endoscopy units [17,18], unsafe injection practices performed on medication multi-dose vials or single-dose vials used in multiple patients, accounted for the respective nosocomial outbreaks.

In the present report, the transmission of HCV in an outbreak involving three patients attending an endoscopy unit is studied. Two of these patients tested HCV positive shortly after undergoing colonoscopy. An epidemiological investigation was combined with the use of molecular biology techniques and genetic and phylogenetic analyses to identify the origin of the outbreak and the transmission route. This reveals the importance of viral genetic variability estimates as a valuable complement to epidemiological search.

## Results

### Epidemiological Investigation

**Figure 1:**
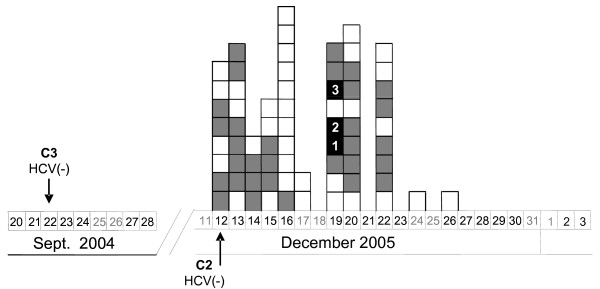
**Chronological order of endoscopy procedures by date**. Chronological scheme of the group of patients analyzed. Boxes represent endoscopy procedures performed to patients who attended the diagnostic unit. Patient order is deduced from the bottom to the top of the column. White boxes correspond to gastroscopies; grey boxes to colonoscopies and black boxes to colonoscopies and outbreak cases. Arrows indicate the date of the last negative HCV-RNA test for patients C2 and C3. It can be deduced from the columns that the order in which endoscopies performed on 19^th ^December 2005 was: gastroscopy, gastroscopy, colonoscopy, patient C1 colonoscopy, patient C2 colonoscopy, gastroscopy, patient C3 colonoscopy, colonoscopy and colonoscopy.

On February 2006, two regular blood donors were independently diagnosed with acute HCV infection. Both individuals had undergone colonoscopy procedures at the same endoscopy unit on December 19^th^, 2005. These two patients (C2 and C3) had tested negative for HCV prior to colonoscopy. Thus, the time of putative transmission was considered to be comprised between the last HCV negative test of these patients and the dates of their onset of symptoms (Figure 1 and Table 1). The initial wide case-finding investigation focused on recorded HCV status of patients attending the colonoscopy unit from July 2005 to February 2006 and did not reveal any suspect of nosocomial infection. Next, a more limited and detailed case-finding search was carried out among all attendants to the same endoscopy unit between December 12^th^, 2005 and December 26^th^, 2005. Apart from the two index cases, 39 patients matched temporal criteria, but three were excluded due to exitus. The active search for HCV-infected cases among these 36 eventually exposed patients (30 of which had gone through colonoscopy and the remaining 6 through gastroscopy) revealed two asymptomatic HCV-positive individuals (C1 and C4). The epidemiological questionnaire indicated that patient C1, but not patient C4, had also undergone a colonoscopy on the same day as patients C2 and C3.

**Table 1:** Epidemiological data.

Patient	C1	C2	C3
**Type of case**	Prevalent	Incident	Incident
**First symptoms**	Asymptomatic	17/01/2006	26/01/2006
**HCV-negative test**	No	12/12/2005	22/09/2004
**HCV subtype**	1b	1b	1b
**Previous risk factors**			
**Contact with HCV (3 years)**	No	Yes*^a^*	No
**Invasive diagnose or treatment**	Colonoscopy (2001)	Acupuncture (Feb.-July 2005)	No
**Surgery**	Caesareas (1984 and 1987)	No	No

**Risk factors at the endoscopy unit**			
**Admission day**	19/12/2005	19/12/2005	19/12/2005
**Blood collection and intravenous line insertion: order**	1^st^	3^rd^	2^nd^
**Sedation and colonoscopy: order**	1^st^	2^nd^	3^rd^
**Biopsy**	No	No	Yes*^b^*

Patient report and nosocomial risk factors

*^a ^*Contact with HCV-positive patient.

*^b ^*Polypectomy.

HCV subtypes for patients C1, C2 and C3 were 1b, while that of C4 was subtype 2c. This patient was considered to be unrelated to the outbreak and was removed from further analyses. Relevant features and nosocomial risk factors of patients C1, C2 and C3 are shown in Table 1. On December, 19^th^, 6 other patients had undergone endoscopy diagnosis (3 gastroscopies and 3 colonoscopies) (Figure1). Data extracted from epidemiological questionnaires (Table 1) strongly indicated that patient C1 was the likely source of infection of the two incident cases. One additional patient underwent gastroscopy between colonoscopies practiced on patients C1 and C2 (Figure 1) but tested negative for HCV.

The epidemiological investigation focused on determining the transmission route. The procedures that could have led to patient-to-patient infection were examined: (a) blood collection and peripheral intravenous line placement and (b) sedation in the endoscopy room, later followed by colonoscopy. In the former, usual practices and decision-making about materials largely depend on the nurse in charge. Patient-nurse assignment was not recorded although it could be traced after interviews, and written protocols were not available. In spite of this, interviews to nurses did not reveal any failure in disinfection standards applied to blood-related practices.

The same anaesthesiologist, endoscopist, two nurses and auxiliary nurse performed anesthesia, colonoscopy procedure and nurse assistance, respectively, on all patients that underwent endoscopies on December 19^th^, including patients C1, C2 and C3. Written records documented that these three patients received parentally midazolam, propofol and anfentanil for anesthetic purposes. The patient that underwent gastroscopy between colonoscopies practiced on patients C1 and C2 received propofol and lidocaine. It could not be checked whether medication vials were multi- or mono-dose and whether they were used on more than a patient. Written protocols for sedation were not available. Interviews of medical personnel related to sedation procedures did not reveal any failure in preventive measures of infection.

The endoscopy unit had two colonoscopes, for which traceability failures were detected in patient assignment and disinfection records. A registered processing label recovered from the supply unit of endoscopes along with data from questionnaires established that patient order in sedation and colonoscopy was C1, C2 and C3 (Table 1). Biopsy specimens were obtained only from patient C3 by means of sterilized forceps provided by the central sterilization unit. Although it was not possible to verify whether the same colonoscope had been used with patients C1, C2 and C3, examination of the endoscope disinfection routines could not rule out this possibility. General standard guidelines for decontamination included manual cleaning of equipment followed by automatic disinfection. According to the available written protocol, obstruction of colonoscope channels with gastrointestinal debris could not be ruled out completely. In this procedure, colonoscope channels were flushed and rinsed with water and externally washed. After removing the valves, colonoscopes were immersed in enzymatic detergent and brushed manually. Individual clearance of all colonoscopy channels was not checked. Next, channels were rinsed with cleansing solution and introduced into the automatic-washer disinfector. As for the automatic step, correct disinfection of colonoscopes could not be checked due to a failure of the printer connected to the disinfection device. In addition, technical staff members in charge of colonoscope disinfection had not received specific training.

### Phylogenetic analyses and viral variability

**Figure 2:**
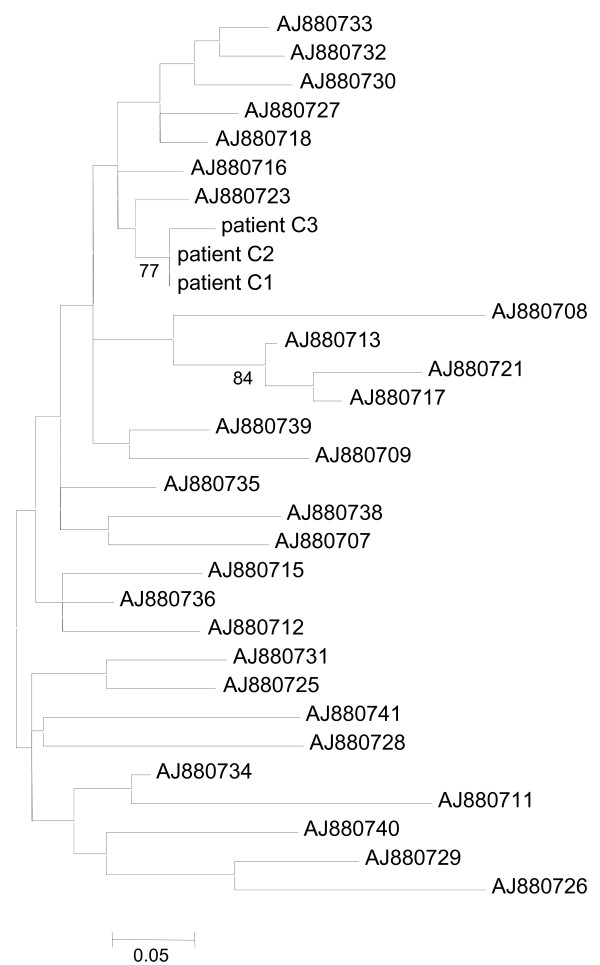
**Maximum-likelihood phylogenetic tree obtained for the NS5B region**. Maximum-likelihood phylogenetic tree obtained for the NS5B region of patients analyzed in this study (C1, C2 and C3) and 28 reference sequences. All sequences belong to HCV subtype 1b. Only bootstrap support values higher than 70% are indicated. The scale bar represents genetic distance.

Partial NS5B sequences obtained from patients C1 and C2 were identical and only differed in three of 337 positions from the sequence corresponding to patient C3. As a result, the phylogenetic tree of the NS5B region (Figure 2) showed that patients C1, C2 and C3 clearly clustered in a monophyletic group among sequences belonging to HCV subtype 1b (bootstrap support 77%). The analysis of the NS5B region clearly defined the extent of the outbreak. As expected in a well-defined outbreak, the genetic distances between sequences belonging to patients C1, C2 and C3 were substantially shorter than those between them and local reference sequences from epidemiologically unrelated patients (Figure 2). In addition, another well-supported cluster also appeared among the reference sequences probably indicating local epidemiological links between three isolates in the past. Monophyletic clustering of sequences in the phylogenetic tree of the conserved NS5B region enabled us to define the patients involved in the outbreak. In order to further characterize the respective HCV viral populations, the highly variable E1-E2 region of HCV was analysed.

Thus, sequences from 74 cloned fragments of the E1-E2 region, encompassing the hypervariable region (HVR), were obtained from patients C1, C2 and C3 (26, 28 and 20 sequences respectively). In order to avoid redundancy, only unique sequences within each patient were used in phylogenetic analysis. Therefore, a total of 38 sequences (21, 15 and 2 unique sequences from patients C1, C2 and C3, respectively) were included in the phylogenetic analysis.

**Figure 3:**
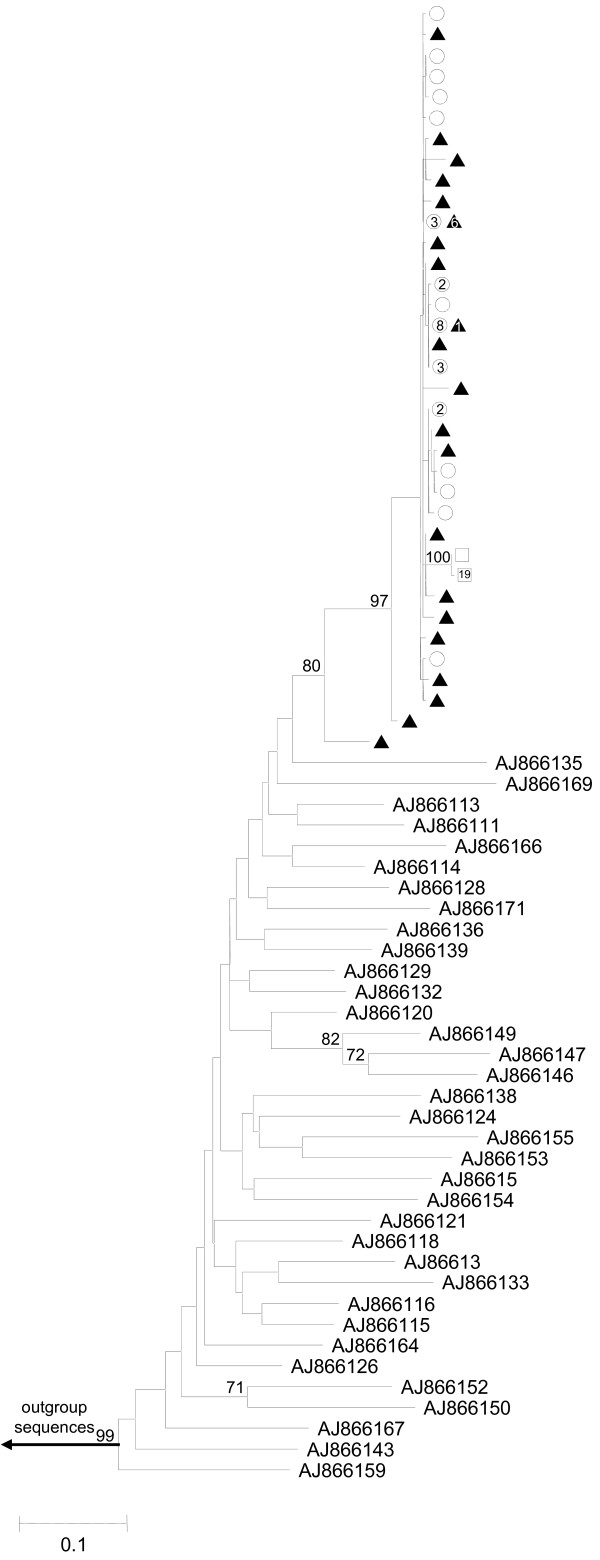
**Maximum-likelihood phylogenetic tree obtained for the E1-E2 region**. Maximum-likelihood phylogenetic tree obtained for the E1-E2 region of cloned sequences from patient C1 (black triangles), patient C2 (white circles), patient C3 (white squares) and 35 reference sequences. Numbers inside shapes indicate number of identical sequences sampled. All sequences belong to HCV subtype 1b. Only bootstrap support values higher than 70% are indicated. The scale bar represents genetic distance.

These E1-E2 cloned sequences were analysed along with 35 local reference sequences from epidemiologically unrelated patients. The resulting phylogenetic tree, in which only different sequences within patient were included, is shown in Figure 3. Sequences of clones from patients involved in the outbreak gave rise to a well-supported monophyletic group (bootstrap support 80%). Most of the 21 different cloned sequences obtained from patient C1 were unique and showed relatively short distances from each other. However, two divergent sequences were also present among the isolated cloned sequences. Patient C2 provided 15 different cloned sequences, some of which were repeatedly sampled between 2 and 8 times. All these sequences mixed with those from the main cluster of patient C1 sequences in the phylogenetic tree (Figure 3). Moreover, two sequences from patient C1 were also detected in patient C2 (one was sampled three times and the other eight times). Finally, patient C3 presented two unique cloned sequences which grouped in a differentiated cluster (bootstrap support 100%) also located among sequences of the main cluster of patient C1 sequences, with one sequence sampled 19 times and the other only once (Figure 3). In congruence with the phylogenetic tree of the NS5B region, genetic distances between E1-E2 sequences from patients C1, C2 and C3 were much shorter than those between these and the reference sequences.

The outbreak pattern in phylogenetic trees, i. e. monophyletic cluster of outbreak-related sequences, is obvious in both NS5B and E1-E2 regions. However, due to the larger number of mutations accumulated in the more variable E1-E2 region, genetic distances in this portion of the genome can provide additional information on how viral populations have emerged in patients involved in the outbreak. Two additional well-supported clusters appeared among the reference sequences for the E1-E2 region (Figure 3), probably indicating local epidemiological links involving two and three isolates, respectively.

Intrapatient genetic variability for the E1-E2 region estimates (Table 2) showed the highest variability for patient C1 viral population in all parameters (haplotype diversity, number of polymorphic sites, total number of mutations, nucleotide diversity and average number of nucleotide differences between pairs of sequences) followed by lower values for patient C2 and the lowest genetic variability corresponding to patient C3. For instance, nucleotide diversity (π) of patient C1 viral population was one and two orders of magnitude larger than those of patients C2 and C3, respectively. Therefore, these estimates showed a pronounced decrease in genetic variability from patient C1 to patient C3 (C1>C2 > C3). In a reconstructed scenario, first, a viral inoculum from patient C1 was in contact with patient C2 and later with patient C3. An unidentified factor could have reduced the remaining viral population after infecting patient C2, but left enough viral load to cause infection in patient C3.

**Table 2:** Summary of intrapatient genetic variability for the E1-E2 region of HCV-1b analyzed in this study

Patient	n^a^	h^b^	Hd^c,h^	S^d^	η^e^	π^f,h^	κ^g,h^
**C1**	26	21	0.954 (0.035)	78	79	0.01698 (0.00567)	9.034 (4.300)
**C2**	28	15	0.905 (0.042)	17	17	0.00622 (0.00083)	3.310 (1.753)
**C3**	20	2	0.100 (0.088)	1	1	0.00019 (0.00017)	0.100 (0.179)

^a^n, number of clone sequences.

^b^h, number of different haplotypes.

^c^Hd, haplotype diversity.

^d^S, number of polymorphic sites.

^e^η, total number of mutations.

^f^π, nucleotide diversity.

^g^κ, average number of nucleotide differences between pairs of sequences.

^h^Standard deviations are reported in parentheses.

## Discussion

In the present study, an approach combining epidemiological investigation with a molecular strategy involving both phylogenetic and genetic variability characterization was implemented to ascertain the most likely explanation for two iatrogenic infections. This report of an outbreak of two acute HCV among three outpatients, attending an endoscopy unit on the same day, provides a case study of nosocomial HCV transmission. Phylogenetic analyses using NS5B and E1-E2 viral regions clearly defined the extent of the outbreak.

The fact that an HCV negative patient had undergone gastroscopy between colonoscopies practiced between patient C1 and C2 along with the fact that all three HCV-infected patients underwent colonoscopy, led the investigation to consider colonoscopy, and not gastroscopy, as a risk factor for the outbreak. Hence, common procedures practiced before gastroscopies and colonoscopies, although fully examined, were considered not to have played a relevant role in the patient-to-patient transmission.

Therefore, nosocomial infection occurred during colonoscopy procedure, but the recovered epidemiological data could not clearly settle whether the transmission resulted from eventual contamination of medication vials or incomplete disinfection of colonoscopes. The lack of information related to vials used in sedation and the lack of traceability in the use of colonoscopes have limited our ability to detail how transmission occurred. A similar dilemma could not be resolved in the pioneering case report of HCV transmission during colonoscopy [11] in which the authors were unable to completely rule out the possibility that HCV transmission occurred during the anaesthesia procedure previous to the colonoscopy. Similar uncertainty was also expressed in other case reports studying HCV transmission during colonoscopy diagnosis [19,20]. However two recent reports have successfully solved that unsafe injection practices performed with medication vials accounted for nosocomial infections [17,18]. In fact, several studies have shown that unsafe injection practices related to anesthesia or intravenous medication are more often involved in HCV and HBV transmission than the equipment used in medical procedures with the patients [10,18]. In this respect, reuse of single or multiple-dose medication vials on multiple patients appears as the most common risk factor associated to minor invasive procedures such as colonoscopies [17,18]. The strict adherence to disinfection standards on diagnosis instruments and the application of safe injection practices fully described elsewhere [10,17,18,20] would prevent such nosocomial patient-to-patient infections definitively.

It has often been observed that, in the absence of treatment, a viral population from chronically infected patients tends to gradually accumulate genetically diverse viral variants over time [21,22]. On the other hand, a viral population from recently infected patients tends to display lower genetic diversity than the viral population from the source [23]. Although viral load of inoculums, transmission pathway or host factors, such as susceptibility to infection or a particular immune system response, may play a role in the emergence of viral populations, the pattern of decreasing variability of viral populations from source patient to newly infected patient should be met. Virus sequences isolated from patient C1 displayed the highest intrapatient variability in the E1-E2 region, which is a feature of the oldest infection. Intrapatient variability was lower in patient C2, while in patient C3 it was minimal, thus matching the order in which they underwent sedation and colonoscopy. A similar relationship has already been observed in other nosocomial transmission case of HCV (Bracho, unpublished results). As the exact route of transmission could not be ascertained, two alternative hypothesis of progressive viral load reduction of the infective inoculums can be formulated. If infection occurred via contaminated medication, initial infective virions from patient C1 could have undergone progressive deterioration in an inappropriate media so that patient C3 received a much more limited representation of the diversity present in the source viral population than the previously infected patient (i.e. patient C2). If infection occurred via colonoscope, the colonoscope contaminated with virions from patient C1 could have undergone two incomplete disinfection cycles. This procedure, performed after colonoscopy practiced on patient C1 and later on patient C2, could have reduced the viral population in the colonoscope progressively. Therefore, independently of the true route of transmission, a reduction in viral diversity is predicted under both scenarios.

Thus, these results, in congruence with but independently of the epidemiological investigation, support that patient C1 was the source of this outbreak and that it is highly likely that infection to patients C2 and C3 occurred during the colonoscopy procedure. A typical pattern in phylogenetic trees, the monophyletic clustering of outbreak-related sequences with high bootstrap support, is often recognized when using consensus sequences of cases and controls from either conserved (core, NS5B) or variable regions (E1-E2) of the HCV genome. These sequences are obtained through direct sequencing of PCR products and their use is limited to determining which patients are involved in the putative outbreak [12,24-27]. Mutations accumulate in viral populations mainly as a function of time and evolutionary rate, which is substantially larger for hypervariable regions [28,29]. Hence, genetic distances are larger in variable regions than in more conserved ones and, when calculated for intrapatient viral populations, provide a useful tool for relating genetic variability to the time elapsed since infection. This is especially the case in the characterization of recent transmissions [23]. As a consequence, accurate determination of the infection source, especially in rapidly detected outbreaks, is best tackled by the phylogenetic analysis of individual (cloned or end-point diluted) sequences of a fast evolving region [17,18,30] and complemented with genetic variability estimation of the intrapatient viral populations [23]. The combination of these two procedures will improve the resolution of molecular studies of outbreaks over that of analyses based only on consensus sequences.

## Conclusions

Based on examination of data collected by an epidemiological investigation, and genetic and phylogenetic results, we conclude that patient-to-patient transmission probably occurred sequentially during colonoscope procedures. The exact route of transmission, putatively through intravenous medications or colonoscope, could not be determined. The comparison of intrapatient viral genetic variability estimates is proposed as a powerful tool for tracing HCV transmission. As prevention of nosocomial transmissions should be a prime concern, our study contributes to reinforce the importance of implementing good practices in invasive diagnosis.

## Methods

### Epidemiological investigation

The newly HCV positivity of the two index patients after undergoing colonoscopy in a private endoscopy unit on 19^th ^December, 2005 lead to a retrospective survey to study a possible nosocomial transmission. First, a wide case-finding investigation included reviewing HCV-positive and negative records from all local Microbiology Units between January 2004 and February 2006 (eight hospitals, including the one under study) of patients that had undergone endoscopy procedures between July 2005 and February 2006 at the endoscopy unit. A second, more limited and detailed search involved all patients who had attended the endoscopy unit one week before and after the date considered. This investigation covered review of clinical records for 39 patients matching temporal criteria. All these patients, except three due to exitus, were actively contacted, informed about the possibility of exposure to HCV and tested for anti-HCV. The 36 patients completed a detailed epidemiological questionnaire on risk factors for HCV infection including history of transfusion, intravenous drug use, piercing, tattooing, acupuncture and suspicion of nosocomial transmission. In order to rule out delayed seroconversion, the six patients that had undergone colonoscopy or gastroscopy on 19^th ^December, 2005 and tested negative for HCV in February 2006, were retested 6 months later. All of them were found to be anti-HCV negative at that time. Healthcare staff involved in performing endoscopy (one gastroenterologist, one anesthesiologist, seven nurses and one nurse's aide) had negative HCV status in February 2006.

An epidemiological investigation to determine risk procedures leading to HCV transmission during endoscopies was also carried out. This included a description of the facilities, review of clinical practices and procedures performed between admission and discharge, description of staff member qualifications and assessment of disinfection procedures and devices (i.e. observation of endoscopic procedures and endoscopic reprocessing, and observation of procedures regarding intravenous line insertion for anaesthetic purposes). All healthcare workers were interviewed about infection-control practices.

### Patients and specimens

Serum samples were obtained from four HCV-infected patients (C1, C2, C3 and C4) referred to the endoscopy unit. Samples were taken on February 2006 and stored frozen at -70°C until processed. Viral load of specimens was not measured.

### RNA extraction and RT-PCR of NS5B and E1-E2 regions

Viral RNA was obtained from 200 μl of serum from each sample using a High Pure Viral RNA Kit (Roche Diagnostics GmbH, Mannheim, Germany). Reverse transcriptions (RT) and PCRs were performed as described in Bracho *et al*. (2005) [23]. Amplified fragments were 337 and 532-nucleotide-long for NS5B and E1-E2 regions, respectively. Oligonucleotides used for amplification and direct sequencing of the NS5B region were 5'-TATGATACYCGCTGYTTYGACTC-3' (sense), 5'-GTACCTRGTCATAGCCTCCGTGAA-3' (antisense), and for the amplification of the E1-E2 region, 5'-CGCATGGCYTGGGAYATGAT-3' (sense), 5'-GGGGTGAARCARTAYACYGG-3' (antisense), and hemi-nested 5'-GGGATATGATRATGAAYTGGTC-3' (sense). A single amplified product for each region was observed after electrophoresis on a 1.4% agarose gel stained with ethidium bromide. Amplified products of the NS5B and E1-E2 regions were purified with High Pure PCR Product Purification Kit (Roche Diagnostics GmbH, Mannheim, Germany). The NS5B gene sequences were obtained directly with the ABI PRISM BigDye^® ^Terminator v3.1 Cycle Sequencing Kit in an ABI 3730 (Applied Biosystems Foster City, CA) automated sequencer and amplification oligonucleotides as described in Bracho et al. (2005).

### Cloning and sequencing of the E1-E2 region

Amplified fragments of the E1-E2 region, encompassing HVR1, HVR2 and HVR3 [22], were directly cloned in EcoRV-digested pBluescript II SK (+) phagemid (Stratagene, La Jolla, CA). Plasmid DNA was purified with a High Pure Plasmid Isolation kit (Roche). Recombinant plasmids were sequenced with KS and SK oligonucleotides (Stratagene) as described above.

### Phylogenetic and genetic analyses

Partial NS5B sequences were genotyped using HCV BLAST search at Los Alamos HCV Sequence Database [31]. NS5B sequences from patients C1, C2, and C3 were analyzed along with a panel of 28 additional local sequences from epidemiologically unrelated isolates [23], including NS5B reference sequences for HCV subtype 1b. Accession numbers for the NS5B reference sequences (EMBL) are shown in Figure 1. Cloned sequences of the E1-E2 region, corresponding to the three patients infected with HCV subtype 1b, were analyzed along with 35 reference sequences of HCV subtype 1b. These E1-E2 reference sequences were also obtained from patients included in the above study [23]. The accession numbers for E1-E2 region reference sequences are shown in Figure 2. Prior to phylogenetic analysis of the E1-E2 region, unique cloned sequences within patient were selected using DAMBE software [32]. For both HCV genome regions, multiple sequence alignments were obtained using ClustalX 2.0.10 [33]. Phylogenetic trees were constructed by maximum likelihood with PhyML v3.0 [34] using the nucleotide substitution model that best fitted the data according to corrected Akaike Information Criterion (AICc) implemented in jModeltest 1.0.1 [35]. The evolutionary model that best explained the data in the NS5B region corresponded to TIM2 model distance [35], with a gamma distribution accounting for heterogeneity in evolutionary rates among sites (shape parameter = 1.034) and a proportion of invariable sites (I = 0.630). The evolutionary model that best explained the data for the E1-E2 region corresponded to Tamura-Nei [36], with a gamma distribution accounting for the heterogeneity in evolutionary rates among sites (shape parameter = 0.737) and a proportion of invariable sites (I = 0.273). In addition, a parallel analysis was carried out with E1-E2 cloned sequences from local reference patients instead of the corresponding consensus sequences, but the maximum likelihood tree yielded similar results (data not shown). Robustness of the clades derived in the phylogenetic trees was assessed by bootstrap analysis using 1000 pseudo-replicates as implemented in PhyML. Genetic variability estimates for the complete set of E1-E2 cloned sequences derived from patients C1, C2 and C3 were obtained with DnaSP v5 [37].

### Nucleotide sequence accession numbers

The HCV sequences obtained in this study have been deposited in GenBank under accession numbers [EMBL:FM881903 to EMBL:FM881906] for NS5B region sequences and [EMBL:FM955149 to EMBL:FM955222] for clones of the E1-E2 region.

## Competing interests

The authors declare that they have no competing interests.

## Authors' contributions

SG, RC, HV and FG performed epidemiological investigation. AV made contribution to the sequence generation and submission, and drafted tables and figures. MAB and FG-C carried out the molecular genetic studies and wrote the manuscript. HV and FG-C coordinated the study. SG, RC, HV, FG, MAB and FG-C participated in the interpretation of data and critically revised the manuscript. All authors read and approved the final manuscript.

## References

[B1] Rantala M van de Laar MJ Surveillance and epidemiology of hepatitis B and C in Europe - a review Euro Surveill 2008 13 18761967 10.2807/ese.13.21.18880-en

[B2] Rosen HR Primer on hepatitis C for hospital epidemiologists Infect Control Hosp Epidemiol 2000 21 229 234 10.1086/501752 10738998

[B3] Prati D Transmission of hepatitis C virus by blood transfusions and other medical procedures: a global review J Hepatol 2006 45 607 616 10.1016/j.jhep.2006.07.003 16901579

[B4] Shepard CW Finelli L Alter MJ Global epidemiology of hepatitis C virus infection Lancet Infect Dis 2005 5 558 567 10.1016/S1473-3099(05)70216-4 16122679

[B5] Sy T Jamal MM Epidemiology of hepatitis C virus (HCV) infection Int J Med Sci 2006 3 41 46 16614741 10.7150/ijms.3.41 PMC1415844

[B6] Esteban JI Sauleda S Quer J The changing epidemiology of hepatitis C virus infection in Europe J Hepatol 2008 48 148 162 10.1016/j.jhep.2007.07.033 18022726

[B7] Global surveillance and control of hepatitis C. Report of a WHO Consultation organized in collaboration with the Viral Hepatitis Prevention Board, Antwerp, Belgium J Viral Hepat 1999 6 35 47 10.1046/j.1365-2893.1999.6120139.x 10847128

[B8] Irving WL Salmon D Boucher C Hoepelman IM Acute hepatitis C virus infection Euro Surveill 2008 13 18761963 10.2807/ese.13.21.18879-en

[B9] Zeuzem S Teuber G Lee JH Ruster B Roth WK Risk factors for the transmission of hepatitis C J Hepatol 1996 24 3 10 8836883

[B10] Alter MJ Healthcare should not be a vehicle for transmission of hepatitis C virus J Hepatol 2008 48 2 4 10.1016/j.jhep.2007.10.007 18023493

[B11] Bronowicki JP Venard V Botte C Monhoven N Gastin I Chone L Hudziak H Rihn B Delanoe C LeFaou A Patient-to-patient transmission of hepatitis C virus during colonoscopy N Engl J Med 1997 337 237 240 10.1056/NEJM199707243370404 9227929

[B12] Bruguera M Saiz JC Franco S Gimenez-Barcons M Sanchez-Tapias JM Fabregas S Vega R Camps N Dominguez A Salleras L Outbreak of nosocomial hepatitis C virus infection resolved by genetic analysis of HCV RNA J Clin Microbiol 2002 40 4363 4366 10.1128/JCM.40.11.4363-4366.2002 12409433 PMC139636

[B13] Ciancio A Manzini P Castagno F D'Antico S Reynaudo P Coucourde L Ciccone G Del Piano M Ballare M Peyre S Digestive endoscopy is not a major risk factor for transmitting hepatitis C virus Ann Intern Med 2005 142 903 909 15941697 10.7326/0003-4819-142-11-200506070-00008

[B14] Martinez-Bauer E Forns X Armelles M Planas R Sola R Vergara M Fabregas S Vega R Salmeron J Diago M Hospital admission is a relevant source of hepatitis C virus acquisition in Spain J Hepatol 2008 48 20 27 10.1016/j.jhep.2007.07.031 17998149

[B15] Karmochkine M Carrat F Dos Santos O Cacoub P Raguin G A case-control study of risk factors for hepatitis C infection in patients with unexplained routes of infection J Viral Hepat 2006 13 775 782 10.1111/j.1365-2893.2006.00742.x 17052278

[B16] Delarocque-Astagneau E Pillonel J De Valk H Perra A Laperche S Desenclos JC An incident case-control study of modes of hepatitis C virus transmission in France Ann Epidemiol 2007 17 755 762 10.1016/j.annepidem.2007.05.007 17728145

[B17] Fischer GE Schaefer MK Labus BJ Sands L Rowley P Azzam IA Armour P Khudyakov YE Lin Y Xia G Hepatitis C Virus Infections from Unsafe Injection Practices at an Endoscopy Clinic in Las Vegas, Nevada, 2007-2008 Clin Infect Dis 2010 51 267 273 10.1086/653937 20575663

[B18] Gutelius B Perz JF Parker MM Hallack R Stricof R Clement EJ Lin Y Xia GL Punsalang A Eramo A Multiple Clusters of Hepatitis Virus Infections Associated With Anesthesia for Outpatient Endoscopy Procedures Gastroenterology 2010 139 163 170 10.1053/j.gastro.2010.03.053 20353790

[B19] Le Pogam S Gondeau A Bacq Y Nosocomial transmission of hepatitis C virus Ann Intern Med 1999 131 794 10577314 10.7326/0003-4819-131-10-199911160-00028

[B20] Muscarella LF Recommendations for preventing hepatitis C virus infection: analysis of a Brooklyn endoscopy clinic's outbreak Infect Control Hosp Epidemiol 2001 22 669 10.1086/503391 11842983

[B21] Cuevas JM Torres-Puente M Jimenez-Hernandez N Bracho MA Garcia-Robles I Wrobel B Carnicer F del Olmo J Ortega E Moya A Gonzalez-Candelas F Genetic variability of hepatitis C virus before and after combined therapy of interferon plus ribavirin PLoS One 2008 3 e3058 10.1371/journal.pone.0003058 18725975 PMC2518109

[B22] Torres-Puente M Cuevas JM Jimenez-Hernandez N Bracho MA Garcia-Robles I Wrobel B Carnicer F del Olmo J Ortega E Moya A Gonzalez-Candelas F Using evolutionary tools to refine the new hypervariable region 3 within the envelope 2 protein of hepatitis C virus Infect Genet Evol 2008 8 74 82 10.1016/j.meegid.2007.10.005 18063425

[B23] Bracho MA Gosalbes MJ Blasco D Moya A Gonzalez-Candelas F Molecular epidemiology of a hepatitis C virus outbreak in a hemodialysis unit J Clin Microbiol 2005 43 2750 2755 10.1128/JCM.43.6.2750-2755.2005 15956393 PMC1151931

[B24] Forns X Martinez-Bauer E Feliu A Garcia-Retortillo M Martin M Gay E Navasa M Sanchez-Tapias JM Bruguera M Rodes J Nosocomial transmission of HCV in the liver unit of a tertiary care center Hepatology 2005 41 115 122 10.1002/hep.20515 15619236

[B25] Germain JM Carbonne A Thiers V Gros H Chastan S Bouvet E Astagneau P Patient-to-patient transmission of hepatitis C virus through the use of multidose vials during general anesthesia Infect Control Hosp Epidemiol 2005 26 789 792 10.1086/502618 16209386

[B26] Panella H Rius C Cayla JA Transmission of hepatitis C virus during computed tomography scanning with contrast Emerg Infect Dis 2008 14 333 336 10.3201/eid1402.060763 18258135 PMC2600211

[B27] Quer J Esteban JI Sanchez JM Otero T Rius C Coll M Cubero M Moreno G Gonzalez A Vaque J Nosocomial transmission of hepatitis C virus during contrast-enhanced computed tomography scanning Eur J Gastroenterol Hepatol 2008 20 73 78 10.1097/MEG.0b013e32825b07b0 18090995

[B28] Cuevas JM Gonzalez M Torres-Puente M Jimenez-Hernandez N Bracho MA Garcia-Robles I Gonzalez-Candelas F Moya A The role of positive selection in hepatitis C virus Infect Genet Evol 2009 9 860 866 10.1016/j.meegid.2009.05.007 19463971

[B29] Gonzalez-Candelas F Bracho MA Moya A Molecular epidemiology and forensic genetics: application to a hepatitis C virus transmission event at a hemodialysis unit J Infect Dis 2003 187 352 358 10.1086/367965 12552418

[B30] Ross RS Viazov S Khudyakov YE Xia GL Lin Y Holzmann H Sebesta C Roggendorf M Janata O Transmission of hepatitis C virus in an orthopedic hospital ward J Med Virol 2009 81 249 257 10.1002/jmv.21394 19107970

[B31] Kuiken C Yusim K Boykin L Richardson R The Los Alamos hepatitis C sequence database Bioinformatics 2005 21 379 384 10.1093/bioinformatics/bth485 15377502

[B32] Xia X Xie Z DAMBE: software package for data analysis in molecular biology and evolution J Hered 2001 92 371 373 10.1093/jhered/92.4.371 11535656

[B33] Larkin MA Blackshields G Brown NP Chenna R McGettigan PA McWilliam H Valentin F Wallace IM Wilm A Lopez R Clustal W and Clustal X version 2.0 Bioinformatics 2007 23 2947 2948 10.1093/bioinformatics/btm404 17846036

[B34] Guindon S Gascuel O A simple, fast, and accurate algorithm to estimate large phylogenies by maximum likelihood Syst Biol 2003 52 696 704 10.1080/10635150390235520 14530136

[B35] Posada D jModelTest: phylogenetic model averaging Mol Biol Evol 2008 25 1253 1256 10.1093/molbev/msn083 18397919

[B36] Tamura K Nei M Estimation of the number of nucleotide substitutions in the control region of mitochondrial DNA in humans and chimpanzees Mol Biol Evol 1993 10 512 526 8336541 10.1093/oxfordjournals.molbev.a040023

[B37] Librado P Rozas J DnaSP v5: a software for comprehensive analysis of DNA polymorphism data Bioinformatics 2009 25 1451 1452 10.1093/bioinformatics/btp187 19346325

